# Spontaneous gastric perforation complicating acute laryngotracheobronchitis in an infant: a novel case report from Nepal

**DOI:** 10.1093/jscr/rjad681

**Published:** 2023-12-19

**Authors:** Nabin Paudyal, Yogendra Devkota, Sanjay Kumar Shah, Barsha Gami, Prakriti Regmi

**Affiliations:** Department of General and Laparoscopic Surgery, Nobel Medical College Teaching Hospital, Morang-04, Kanchanbari, Biratnagar, 56613, Nepal; Department of General and Laparoscopic Surgery, Nobel Medical College Teaching Hospital, Morang-04, Kanchanbari, Biratnagar, 56613, Nepal; Department of Pediatric and Neonatal Surgery, Nobel Medical College Teaching Hospital, Morang-04, Kanchanbari, Biratnagar, 56613, Nepal; Department of General and Laparoscopic Surgery, Nobel Medical College Teaching Hospital, Morang-04, Kanchanbari, Biratnagar, 56613, Nepal; Department of General and Laparoscopic Surgery, Nobel Medical College Teaching Hospital, Morang-04, Kanchanbari, Biratnagar, 56613, Nepal

**Keywords:** croup, infant, spontaneous perforation

## Abstract

Pediatric spontaneous gastric perforation is a rarely encountered condition with poorly understood causal mechanisms. We present a novel case of a two-month-old female infant from Nepal who previously experienced Croup and subsequently developed severe abdominal distention and vomiting. Abdominal X-ray findings confirmed pneumoperitoneum, prompting immediate laparotomy. Intraoperative examination revealed a substantial perforation along the posterior stomach wall, specifically along the lesser curvature. The surgical intervention involved gastrorrhaphy and omentopexy using 3-0 Vicryl sutures, leading to an uneventful postoperative recovery. This case report highlights the critical importance of early and efficient management of spontaneous gastric perforations in infants, emphasizing the need for timely intervention to achieve favorable outcomes. Pediatric spontaneous gastric perforation remains a rare condition, and reporting such cases contributes to our understanding and management of this unusual pathology.

## Introduction

Spontaneous gastric perforation in children, especially beyond the neonatal period, is rare and challenging [1]. This first reported successful case from Nepal explores an unusual instance in a two-month-old female child, where the causes were less clear. We consider factors like respiratory distress, aerophagy, gastric wall ischemia, and acute respiratory tract infection as contributors.

## Case report

A 58-day-old female infant arrived at the Nobel Medical College Teaching Hospital in September 2023 with concerns of respiratory distress persisting for 10 days, no bowel movements in the past 24 hours, sudden abdominal distention lasting 8 hours, and occasional non-projectile, non-bilious vomiting. The mother provided the baby’s medical history.

**Figure 1:**
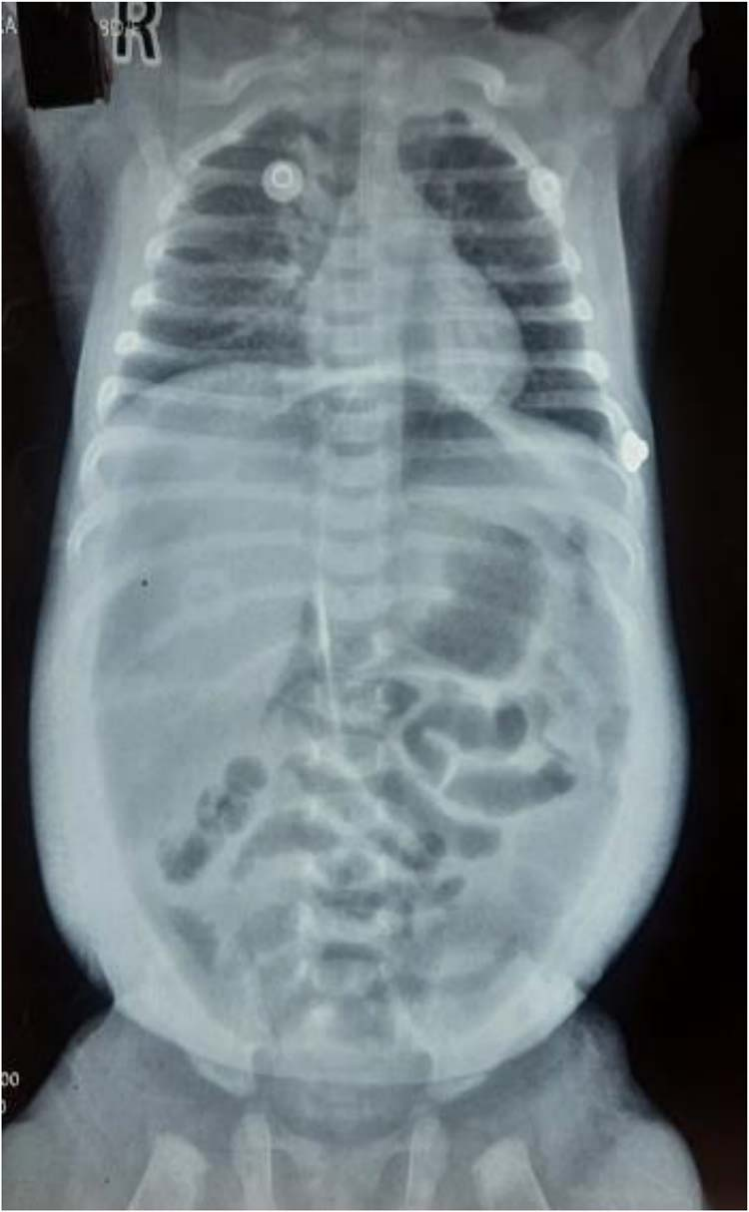
Supine X-ray showing chest and abdomen.

The infant had been previously treated at another facility, where she was admitted in August 2023 due to rhinorrhea, cough, and shortness of breath. She received treatment for Laryngotracheobronchitis (Croup) (Fig. 1) in the pediatric ICU, involving antibiotics, steroids (dexamethasone), nebulization (racemic epinephrine and 3% hypertonic saline), and intravenous fluids. On the sixth day of treatment, she experienced abrupt abdominal distension, leading to nasogastric tube insertion and an immediate referral to our center.

**Figure 2:**
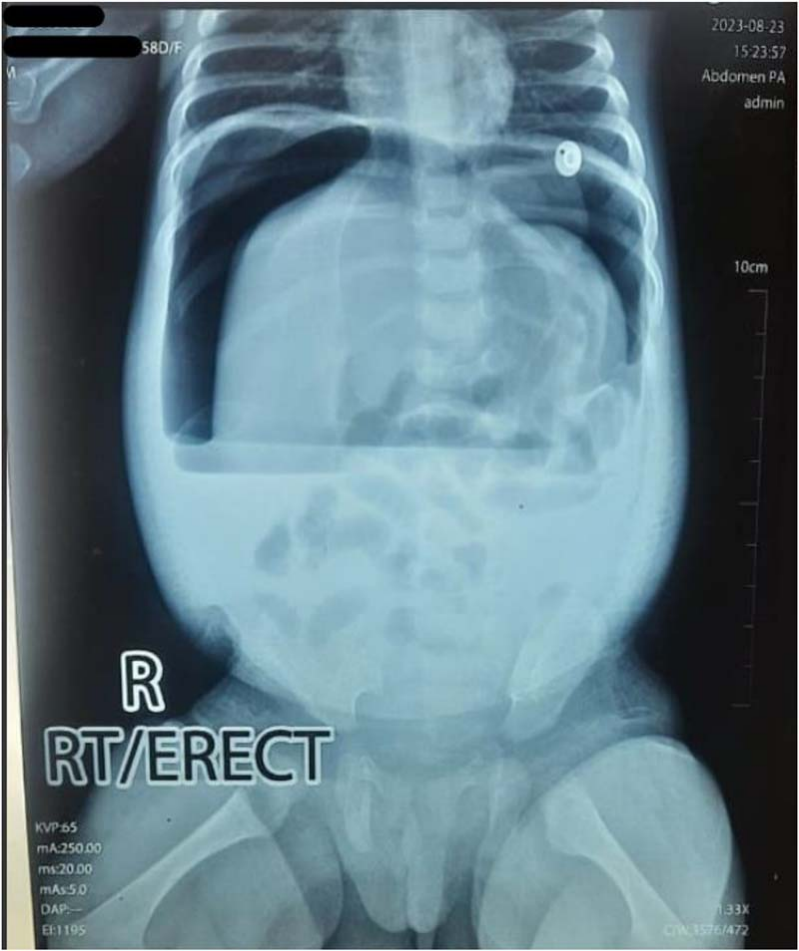
Erect X-ray showing pathological free gas under bilateral dome of diaphragm.

Upon arrival, we conducted a comprehensive medical history review and relevant physical examinations. The baby, delivered via Cesarean section due to fetal distress, had no major health issues except for her mother’s gestational diabetes and a second-trimester urinary tract infection, both managed conservatively. Initial examination indicated signs of dehydration, including a high heart rate, elevated respiratory rate, and 97% oxygen saturation with oxygen support. Abdominal examination revealed distention, visible veins, firmness, and absent bowel sounds. Respiratory and cardiovascular examinations were normal. An erect abdominal X-ray revealed pathological free gas under the right dome of the diaphragm, strongly suggesting a hollow viscus perforation as shown in Fig. 2. Routine blood tests were normal, with a slightly elevated CRP level at 9.5 mg/dl.

Emergency laparotomy was promptly planned after hemodynamic stabilization, with a supraumbilical transverse abdominal incision. Upon entering the peritoneum, a gush of air and 200 ml of bilious collection in the peritoneal cavity were observed. A gastric perforation measuring 2.5 cm × 2 cm with clear edges was identified along the lesser curvature of the stomach in the posterior pre-pyloric region. Gastrorrhaphy using a 3-0 Vicryl suture and omentopexy was performed. Thorough washing and placement of an 18 F abdominal drain within the peritoneal cavity followed. The abdomen was closed using standard techniques.

The baby was transferred to the Pediatric ICU and extubated immediately post-surgery. Postoperative medications included intravenous antibiotics (Piperacillin + Tazobactam, Metronidazole), antihistamines (Ranitidine), acetaminophen, and dextrose. After 2 days of nothing by mouth, we gradually introduced clear fluids and then mother’s milk. On the third postoperative day, the baby experienced three episodes of vomiting, which resolved on its own. She was moved to the ward on the fourth postoperative day, and the abdominal drain was removed on the fifth day once normal feeding resumed. The discharge occurred on the sixth postoperative day, with a follow-up appointment scheduled for 2 weeks later, during which the baby exhibited no significant postoperative issues.

## Discussion

Spontaneous gastric perforation is a rare surgical emergency in children, with a higher incidence among neonates compared to older children. In older children, gastric perforation is usually secondary and can be attributed to factors such as trauma, ingestion of gastro-toxic substances or gastric ulceration [2]. However, spontaneous gastric perforation in children beyond the neonatal period presents distinct characteristics, setting it apart from neonatal cases [1]. In neonates, multiple risk factors have been identified, including prematurity, low birth weight, perinatal asphyxia, exchange transfusion, toxemia of pregnancy, maternal diabetes, placenta previa, chorioamnionitis,or premature rupture of the membranes [3]. In children beyond the neonatal period, risk factors are rarely present. Most of the reported cases of spontaneous perforation have occurred predominantly in female babies of Asian origin. Moreover, the perforation typically assumes a rounded shape and is often located on the posterior wall or the lesser curvature of the stomach similar to our case [4].

Premature birth increases the risk of gastric perforation in neonates due to several factors. These factors include deficiencies in the gastric muscle wall, a lack of intestinal pacemaker cells, and a deficiency in C-KIT mast cells. Gastric smooth cells (SMC) and interstitial cells of Cajal (ICC) share a common early stage in development, with C-KIT expression determining whether they become ICC or SMC. ICC are vital for coordinating stomach movements, including slow rhythmic contractions and neurotransmission throughout the gastrointestinal tract [5].

In premature infants, a reduced number of ICC, due to their underdeveloped state, can lead to decreased gastrointestinal mobility, increasing the risk of spontaneous nasogastric perforation. The immaturity and lack of coordination in esophagogastric motility, common in premature babies, may contribute to higher intragastric pressure, further elevating the likelihood of gastric rupture [6]. A case review done by Albo et al in 1963 including 44 cases of spontaneous gastric ruptures unveiled several consistent features. Notably, a majority of the patients, 34 out of 44, were females, and striking 31 cases displayed ruptures along the lesser curvature of the stomach. The predominance of ruptures at the lesser curvature can be attributed to two primary factors. Firstly, this section of the stomach, known as the magenstrasse, exhibits reduced elasticity due to the absence of mucosal folds. Secondly, when the stomach becomes distended, it tends to assume a spherical shape, subjecting the fixed lesser curvature to greater stretching compared to other stomach regions [7].

In our case, multiple factors could have contributed to the occurrence of gastric perforation. Firstly, the baby had tachypnea due to respiratory distress (Croup), which may have led to gastric wall overdistention and subsequent ischemia, ultimately resulting in perforation due to aerophagy. Additionally, coughing episodes might have led to the ingestion of air, further increasing gastric distention. Secondly, the hypoxemia experienced during the episode of Croup may have exacerbated the ischemic condition, potentially accelerating the perforation. Lastly, the secondary inflammatory response from the gastric mucosa might have also exacerbated the ischemic insult, adding to the complexity of the situation. Furthermore, various scientific studies have shown an association between steroid use and perforation in children, with the inhibition of cytoprotective prostaglandins being the predominant mechanism [8, 9]. In our case, dexamethasone might have been an additional factor responsible for causing the perforation.

Whether omentopexy is needed following gastrorrhaphy remains doubtful. Literature shows either simple gastrorrhaphy alone or gastrorrhaphy with omentopexy with similar results [1, 2, 9]. The choice of procedure may depend upon the discretion of the operating surgeon.

Given the gravity of gastric perforation, swift and precise intervention, combined with the expertise of our dedicated pediatric and anesthetic teams played a pivotal role in ensuring a successful recovery and the baby’s timely discharge from the hospital.

## Conclusion

While spontaneous gastric perforation in children is an exceptionally rare condition, it can be effectively treated with prompt intervention. This inaugural case documented in Nepal underscores the critical significance of early and swift management of spontaneous perforations in infants.
